# Language of written medical educational materials for non-English speaking populations: an evaluation of a simplified bi-lingual approach

**DOI:** 10.1186/s12909-019-1846-x

**Published:** 2019-11-11

**Authors:** Tamim Alsuliman, Lugien Alasadi, Angie Mouki, Bayan Alsaid

**Affiliations:** 1 Service d’Hématologie, Centre Hospitalier Régional Universitaire de Lille, Lille, France; 2 Service d’Hématologie, Hôpital Saint-Antoine, AP-HP, Sorbonne Université, 75012 Paris, France; 3 Department of Gastroenterology and Hepatology, Al-Mouasat University Hospital, Damascus, Syria; 4 Majkop State Technological University, Faculty of Pharmacy, Maykop, Russian Federation 385000; 5 Laboratory of Anatomy, Department of Anatomy, Histology and Embryology, Faculty of Medicine, University of Damascus, Fayez Mansour Street, Damascus, Syria

**Keywords:** Medical education, Language, Alternative, Hybrid approach, Comprehension

## Abstract

**Background:**

Debates have arisen in various non-English speaking countries over the chosen language of instruction in medical education, whether it has to be the English language or the mother tongue. English-based education supporters argue that English is the leading international language of medicine and research, and a crucial tool for Continuing Medical Education (CME), as well as for students who seek practice abroad. On the other hand, mother-tongue-based medical education supporters present it as a way to endorse communication and comprehension between medical practitioners and health care system users, to bridge the gap between practitioners and the paramedical staff, and to overcome linguistic dualism and the language thinking disparity while studying in another. This study aimed to evaluate one of the simplified bi-lingual approaches in terms of medical-educational-written texts for a non-English speaking population: Arabic speaking medical students in specific.

**Methods:**

1546 Arabic-speaking-medical students from different countries participated in a one-step-interactive-experimental-online test. The test assessed participants’ scientific comprehension of three distinct written paragraphs: The first paragraph used conventional mother tongue (Arabic), the second combined English terminology and simplified mother tongue (hybrid), and the third used an English excerpt (English). Two multiple-choice questions (First question in Arabic, second in English) followed each paragraph. Response time was communicated for each paragraph. Participants were asked to select their favorable method.

Repeated Measures ANOVA models and Paired Samples t-Test were used for statistical analysis.

**Results:**

Participants scored a mean of [0.10] for the Arabic paragraph, [0.72] for the hybrid paragraph, and [0.24] for the English paragraph (*P* <  0.001). Results showed a significantly higher mean of points and correct answers within the fastest time for the hybrid paragraph [0.68] compared to the Arabic [0.08] and English [0.18] paragraphs (P <  0.001). Moreover, 50% of participants preferred the hybrid paragraph over the other two paragraphs.

**Conclusions:**

Taking into consideration the large number of participants and the statistically significant results, authors propose that simplified Arabic combined with English terminology may present a viable alternative method for medical-educational-written texts in Arabic-speaking population.

## Background

Debates have arisen in various non-English speaking countries over the chosen language of instruction in medical education, whether it has to be the English language or the mother language [1–3]. English-based education supporters argue that English is the leading international language of medicine and research, a crucial tool for International scientific communication, and Continuing Medical Education (CME), as well as for attending courses abroad. Finally, medical students who seek practice abroad need to be proficient in the English language [3–5]. However, the usage of mother tongue in medical education has been pronounced as a way to promote communication and comprehension between medical practitioners and health care system users and to bridge the gap between practitioners and the paramedical staff [2]. Additionally, Mother language-based education can help overcome linguistic dualism and the language thinking disparity while studying in another. Furthermore, it establishes continuity with the basic education system which is commonly taught in mother tongue [3, 5]. Cost effectiveness represents a complicated discussion point, as changing to mother-language-based-medical education methods may augment the costs of pedagogical materials because of the need to create two-way translation methods, in addition to the cost of training lecturers to use these materials [6, 7].

Medical institutions in Arabic speaking countries are demonstrative examples of a variety of educational methods, specifically in the realm of non-English speaking populations. The majority of medical schools -in Saudi Arabia and Egypt- for example, have curricula that depend entirely on English in contrast to the Arabic based medical curricula that have been adopted for medical education in Syria since the mid-twentieth century with a classical Arabic-based terminology [8–10]. Considering the growing numbers of pre- and post-graduate Syrian students continuing their higher education abroad [11], an assessment of the efficiency of this 70 year-old strategy, in comparison to other experiences of Arabic speaking populations, as well as to a suggested hybrid model, was needed in order to define the extent of positive and negative effects of each method, and also to explore other potentials.

This study aims to assess the scientific understanding of written medical information among Arabic speaking medical students using three linguistic approaches: Unified Medical Dictionary Arabic-based paragraph [12], combined English and simplified Arabic-based paragraph, and an English-based paragraph.

## Methods

A questionnaire was conducted online on (1546) medical students. The targeted population consisted of students in medicine, dentistry and pharmacy faculties in Syrian universities (private and public state universities). While other Arabic speaking students were allowed to submit the questionnaire, their responses were only used in some analysis as a comparison group. The latest online update available of population of students studying medical sciences in Syrian Universities at the time of survey design (the statistical information document of 2013–2014 released by the Ministry of Higher Education in Syria) is counting 22,861 compatible to inclusion criteria students [13]. Statisticians Borg and Gall [14] recommend a sample of 366 for a population of 7500; which means that in our case it is fair to say that, with 1193 responses from Syrian universities students our sample fulfilled these standards.

Nearly 97% of applicants approved to participate while 3% refused. All withdrawals at any stage were respected and the corresponding participants were excluded. The questionnaire was launched online on 14-June-2018 and was withdrawn on 4-August-2018.

The necessary widespread accessibility of this questionnaire was achieved mainly through the medical team of Syrian Researchers platform on Facebook® and Instagram®.

The questionnaire was tested on several versions of web browsers running on different personal computers, tablets, and smartphones with various software systems.

### Study tools

The questionnaire consisted of a series of 10 Java®/Javascript® pages containing 12 questions, built using Google forms®. The survey was designed to assess the three linguistic approaches for written teaching methods of medical sciences. It evaluated these approaches using three paragraphs: Unified Medical Dictionary Arabic-based paragraph [12], combined English and simplified Arabic-based paragraph, and an English-based paragraph. The scientific content of the paragraphs was selected with the assurance that no contamination from previous reading might happen and that medical students are not in-depth familiar with such specialized texts within their curricula. Subjects related to obesity, type 2 diabetes mellitus, and Asthma [15–20] were selected for paragraph 1, 2, and 3, respectively. A two-stage strategy was applied at the time of study design to evaluate the difficulty and the time consumption of the three paragraphs used in our questionnaire. Ten Arabic speaking individuals were asked to assess the three paragraphs regarding the difficulty level (using a five-level scale) and time needed to process the paragraph (reading, comprehension and answering the related questions). In Each stage appropriate changes were made to the texts and modified texts were re-tested until finally an equal average time span to process each paragraph was achieved with the same difficulty level by the testing group. Two multiple-choice questions (the first in Arabic and the second in English) to assess the respondents’ understanding was presented in the following page of each given paragraph along with a third section dedicated for the estimation of the time needed to answer the previous two questions. Two questions regarding participants’ preferred method and preference to learn in mother tongue were also asked in a separated page.

Each page in the multi-stage questionnaire presented distinct non-repetitive content that served the purpose of the questionnaire.

### Data processing and statistical analysis

The data was secured from external access through various levels of password protection. Once the data was obtained it was quickly imported, unmodified, into a secured Microsoft Excel file, processed and then transformed into SPSS® version 20 (IBM) file for Repeated Measures ANOVA models and Paired Samples t-Test statistical analysis.

### Ethical considerations

Participation in this questionnaire was voluntary. Informed consent was obtained from each respondent on the first electronic page of the questionnaire. Furthermore, blinded processing of information was used to insure the anonymity of participation. Participants’ privacy and voluntary participation were guaranteed and rigorously considered throughout the study course. According to these conditions, responses from participants who refused to participate in data analysis –even after completing the questionnaire- or withdrew at any stage of the study were excluded. All included participants were 18 years or older.

## Results

### Participants’ characteristics

A total of 1504 responses were included: 1250 (83%) of responses were from Syrian universities, while 197 (13%) were from other Arabic-speaking populations studying in Arabic-speaking countries and 57 (4%) were originally from Arabic-speaking students studying in non-Arabic-speaking countries. All participants are native Arabic speakers, 763 (51%) of participants were females and 739 (49%) were males, as summarized in (Table 1).

**Table 1:** Characteristics of (1504) Arabic language speaking includable responders. Online Test, 2018

		Number (%) of Responders
Gender	Male	763 (50.73)
	Female	739 (49.14)
	Undetermined	2 (0.13)
College	Preparatory Year	44 (3)
	Medicine College	984 (66)
	Pharmacy College	285 (19)
	Dentistry College	171 (11)
	Other	20 (1)
University	Syrian universities	1250 (83.1)
	Arabic universities	197 (13.1)
	Foreign universities	57 (3.8)
Educational Qualification	Undergraduate	1313 (87)
	Graduate	97 (7)
	MSc	74 (5)
	PhD	14 (1)
	other	6 (0)
University Average	< 65	66 (4.4)
	66–75	425 (28.3)
	76–85	707 (47)
	> 85	286 (19)
	Undetermined	20 (1.3)
Average Age		21.75 years

### Paragraphs’ test assessment

#### Paragraph 1 (Arabic):

Question 1: 372 (24.7%) answers were correct. Question 2: 607 (40.36%) answers were correct.

Both questions were answered correctly by 165 (11%) participants. The time needed to answer these two questions was less than 1.5 min for 1214 (80.7%) of them.

#### Paragraph 2 (Hybrid):

Question 1: 1274 (85%) answered this question correctly, while for question 2: 1343 (89.3%) answers were correct.

Both questions were answered correctly by 1173 (78%) participants, while 331 (22%) had at least one wrong answer. The number of participants who answered these two questions within less than 1.5 min was 1254 (83.4%).

#### Paragraph 3 (English):

Question 1 (in Arabic): 411 (27.3%) answers were correct. Question 2 (in English): 1260 (84%) answers were correct.

These two questions were answered correctly by 346 (23%) participants. The estimated time was less than 1.5 min for 1117 (74.3%) participants.

**Fig. 1:**
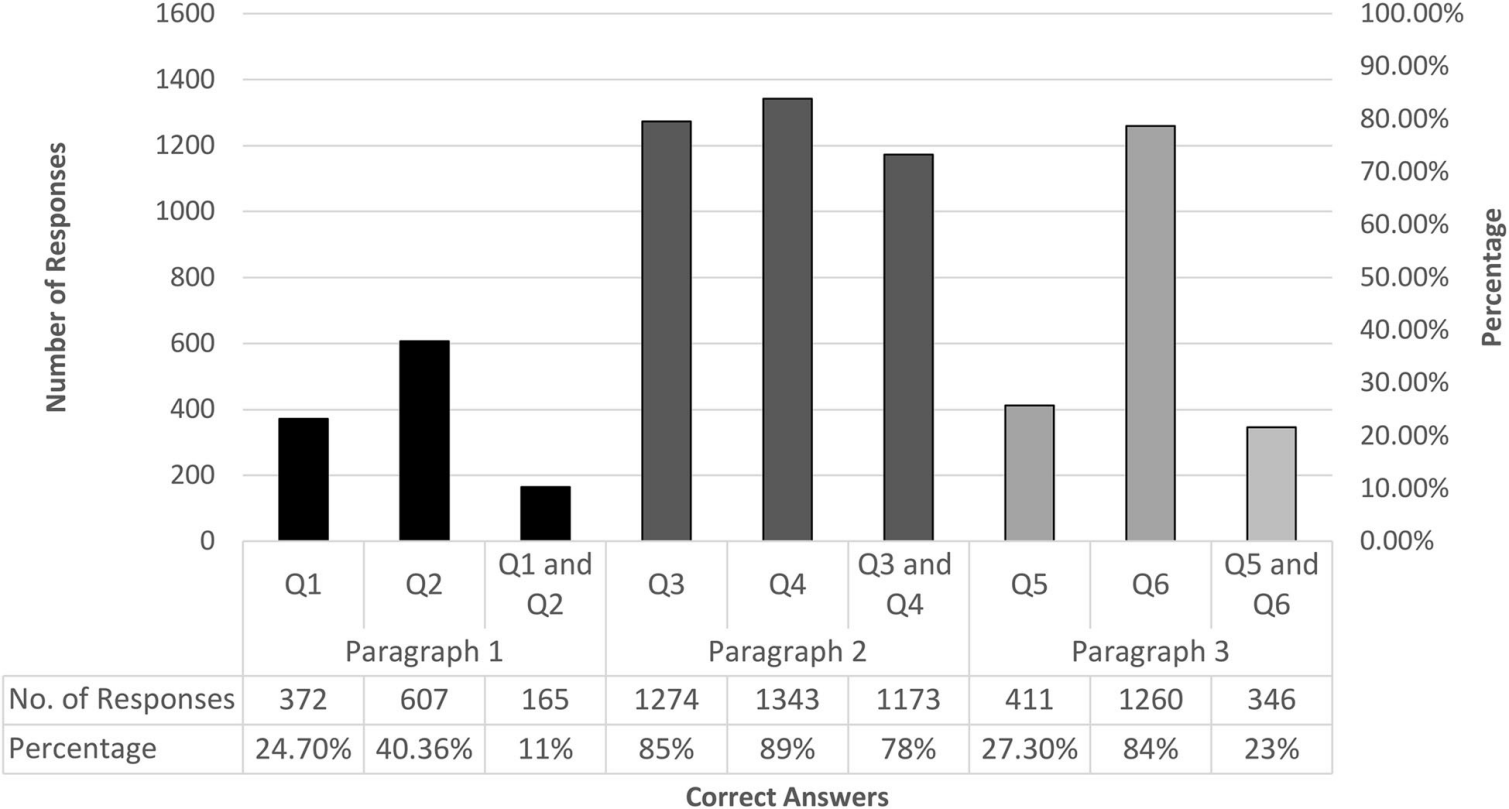
Correct answers distribution among the six questions, two questions per paragraph, and three paragraphs. Results are presented by question for each paragraph then for the sum of each paragraph’s two questions (i.e.: both). Abbreviations: Q: Question. No.: Number

**Fig. 2:**
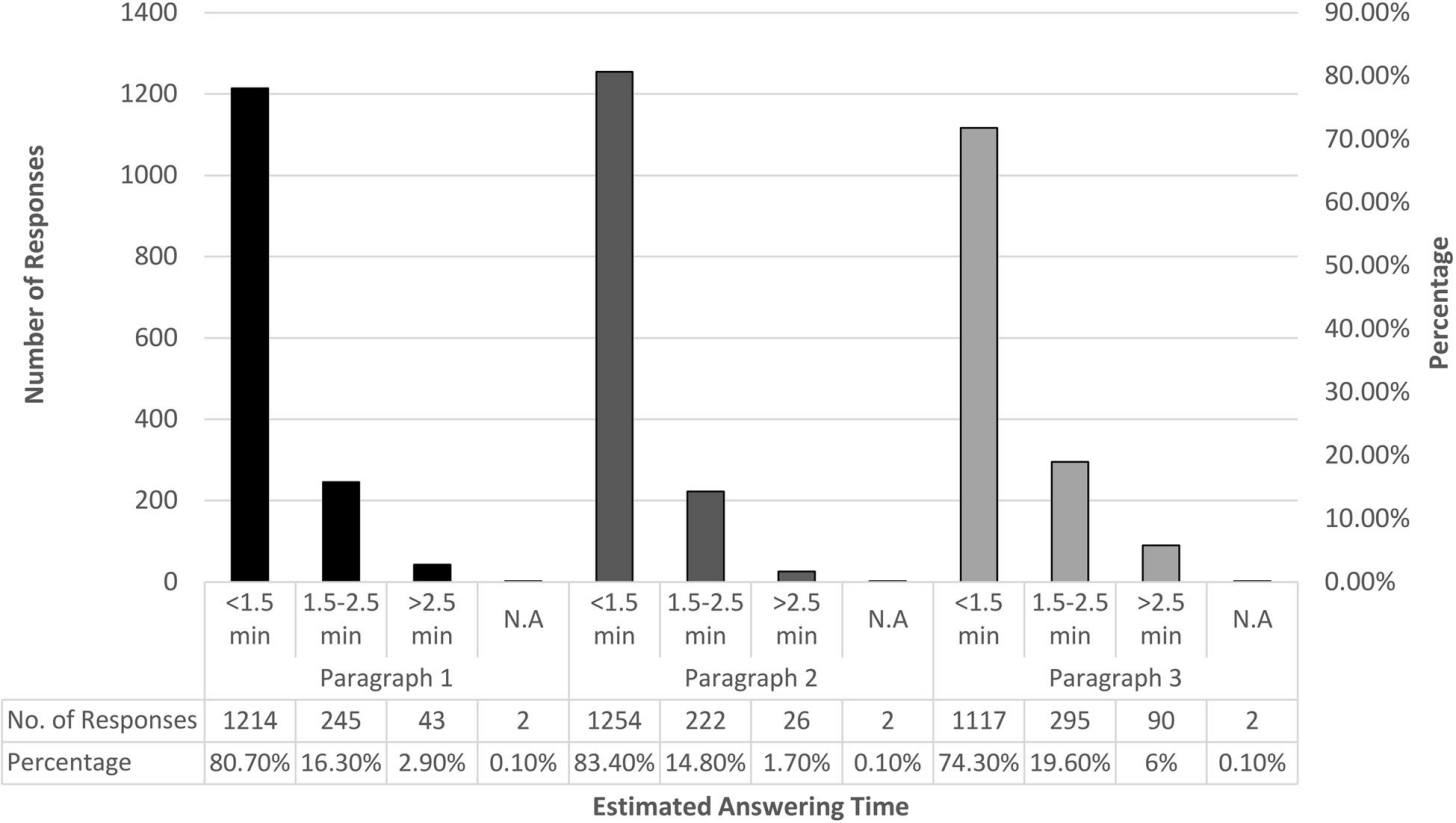
Estimated response time among the three paragraphs. Results are presented per paragraph as time needed to read and respond to the paragraph’s related questions. Abbreviations: Min: Minutes. N.A: Not Available

Results from the three paragraphs are summarized in (Figs. 1and 2 and Additional file 1: Table S1).

The previous data underwent statistical analysis in a three-step process. In order to homogenize the analyzed data, a cohort of 44 (2.97%) responses from preparatory year students and 20 responses with missing data were excluded (Additional file 1: Figure S1).

#### Step 1:

At this stage, only Syrian Universities students’ responses only were included. The total number of valid responses was 1193. Each response with two right answers for one of the three paragraphs was given one point for the corresponding paragraph, and the distribution of points across the three paragraphs was then compared. Paragraph 1 had a mean of [0.10] point. Paragraph 2 had a mean of [0.72] point, while paragraph 3 had a mean of [0.24] point. The difference between the 3 paragraphs was statistically significant with a *P*-value of (< 0.001).

#### Step 2:

Only responses from Syrian Universities students were included with a total number of 1193 responses. One point for each right answer was given, making the potential total points for each paragraph between 0 and 2 points. The mean of points was [0.63] for paragraph 1, [1.75] points for paragraph 2 and [1.13] points for paragraph 3, with a statistically significant difference and a P-value of (< 0.001).

#### Step 3:

**Fig. 3:**
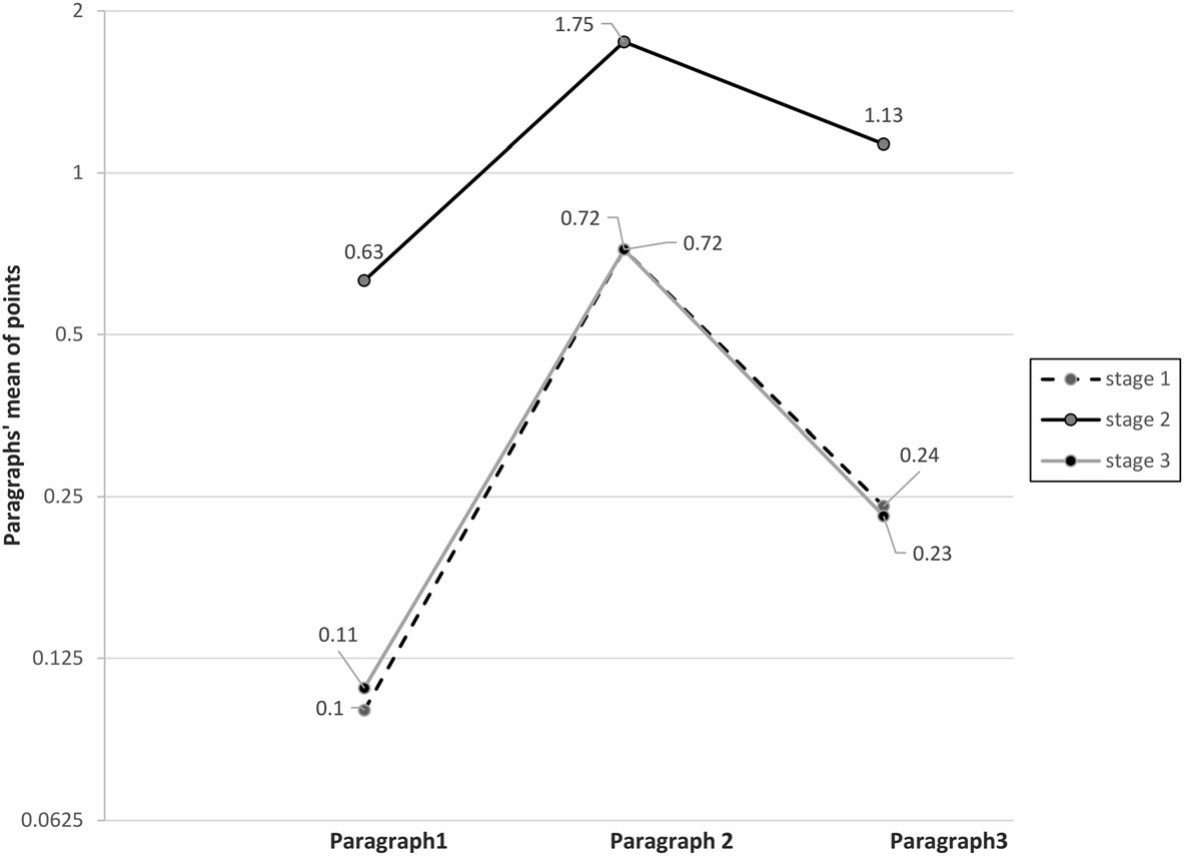
Comparison of the distribution of points for the three paragraphs in the three analytical stages. *Stage 1 (dashed black line):* comparison of points’ distribution of the three paragraphs -one point for each two right answers per paragraph-, all 1193 valid responses from Syrian Universities’ students are included. *Stage 2 (compound black line):* comparison of points’ distribution of the three paragraphs -one point for each right answer per paragraph-, all 1193 valid responses from Syrian Universities’ students are included. *Stage 3 (grey line):* comparison of points’ distribution of the three paragraphs -one point for each two right answers per paragraph-, all 1484 responses from Syrian and non-Syrian Universities; students were included

In order to explore the extensibility of the first two steps on Arabic-speaking populations, responses from Syrian and non-Syrian Universities students were assessed in this stage. The total number of valid responses was 1484. Each response with two right answers for one of the three paragraphs was given one point for the corresponding paragraph. Paragraph 1 had a mean of [0.11] point, while paragraph 2 had a mean of [0.72] point, and it was [0.23] point for paragraph 3, with a statistically significant difference and a *P*-value of (< 0.001). Results from the three stages are summarized in (Fig. 3).

### Comparing correct answers within the shortest (fastest) estimated time between paragraphs

Each response (of the 1193) with response time less than 1.5 min and two right answers was given one point for the corresponding paragraph, while otherwise was given zero. Paragraph 1, 2 and 3 had a mean of 0.08, 0.68 and 0.18 points, respectively, with a *P*-value < 0.001.

### Participants’ preference

In term of preference, 751 (50%) participants preferred the second paragraph’s approach over the other two.

When asked if they prefer medical sciences to be taught in Arabic, 864 (58%) participants answered “yes” while 620 (42%) answered “No”. We found no correlation between Arabic language preference and answering both questions correctly for each of the Arabic, hybrid, and English paragraphs (*P*-value: 0.47, 0.87, and 0.14, respectively). When studying the correlation between the number of correct answers and the language of instruction preference answers, the results showed that the Syrian students who answered both questions for the English-based paragraph right, didn’t prefer Arabic as the language of instruction and that was statistically significant (P-value: 0.012), as shown in Table 2 and Additional file 1: Table S2-S5.

**Table 2:** Distribution of right answers in regard with medical education language preference.^1^ Online Test, 2018

		Number (%) of Responses from Syrian Universities^2^ (total: 1193)			Number (%) of Responses From All Universities^2^ (total: 1440)		
Medical education language preference		Mother language^3^: 737 (62)	Other language: 456 (38)	P-value	Mother language^3^: 864 (58)	Other language: 620 (42)	P-value
Right answers for the first Paragraph		73 (10)	40 (9)	0.51	88 (10)	72 (11.6)	0.47
Right answers for the second Paragraph		576 (78)	359 (79)	0.82	652 (75.4)	477 (77)	0.87
Right answers for the third Paragraph		161 (22)	129 (28)	0.012	182 (21)	154 (25)	0.14
Right answers for all English questions from all paragraphs^4^		217 (29.4)	195 (43)	< 0.001	247 (28.5)	258 (41.6)	< 0.001
Right answers for all mother language questions from all paragraphs^4^		45 (6)	25 (5.4)	0.98	56 (6.4)	38 (6)	0.98
Right answers for all questions		18 (2.4)	11 (2.4)	0.005	24 (2.7)	19 (3)	0.004

^1^Responders were invited to answer if they preferred their mother language for the instruction of medical education or not

^2^Responses from preparatory year not included

^3^Arabic language represents mother language for this population

^4^One question for each of the three paragraphs was in English while the other was in mother language

## Discussion

English is increasingly used as the medium of academia and education across the world [21]. Nowadays, it has occupied a leading role as the international language of medicine and medical publications [4, 22]. Medical students and practitioners need to learn English in order to communicate with other colleagues across the globe, and to attend international conferences and courses [5, 23].

Results showed that both objective and subjective trends towards a hybrid bi-lingual approach for written medical texts among Syrian Universities medical sciences students; this could be justified by the fact that the majority of medical articles are published in English [4], along with accredited references and books. Thus, strictly medical vocabulary, terminology and abbreviations are more common in English, while the non-medical part of the text is usually describing context where normally conducted by the general population and patients in Arabic, so it could be more comprehensible and easy-used in its original language.

The “Language bias” phenomenon is reported as a result of the tendency to publish important findings, especially randomized controlled trials (RCT’s); in English-based journals. The main consequence of this phenomenon is that reviews and meta-analysis that rely on English literature might have a bias towards positive results, and therefore, it may be more common [4].

Furthermore, our findings support what Milosavljević [23] demonstrated in a study conducted at the Medical Faculty of Niš that a significant association between the mother tongue and the English language writing and speaking skills on one side and students’ satisfaction with their social and economic status on the other side.

Our results are also compatible with other findings suggesting that non-English speaking physicians were found to have difficulties comprehending medical literature in strict English, which constitutes a barrier when practicing Evidence-Based Health Care (EBHC) [4, 24].

Nonetheless, learning medical sciences, at least partially, in mother tongue is of particular importance to enhance physician–patient communication; as it is through language physicians can obtain and provide necessary information to their patients [2, 5]. Effective patient-physician communication can improve patient adherence to medical recommendations and overall healthcare outcomes [25]. In a study held in United Arab Emirates (UAE) it was shown that Arabic speaking medical students who received training by means of English-based programs had difficulties in expressing empathy and eliciting patients’ expectations [26]. Another study conducted in Lebanon revealed that the majority of students (88.5%) in Lebanese medical schools were confident they could conduct a medical history in their native language despite receiving their medical education in a foreign language (English or French). Considering the fact that a considerable portion of the Lebanese population is bilingual, and sometimes trilingual; the aforementioned study results cannot be easily generalized to other Arabic language speaking countries [27]. Previous reports have demonstrated that building a successful communication with a patient was challenging when using English medical terminologies by physicians. On the other hand, unifying terminologies enhances the efficiency of interprofessional communication though it may reconsidered as a must [1, 4, 28].

Learning in mother tongue has also been reported to enhance students’ comprehension, communication, retention of information and scientific terms, acquisition of skills, and to reduce the levels of stress [1, 29–31]. Our results showed that a hybrid linguistic approach may be optimal to provide medical written information: simplified Arabic combined with basic English terminology, had experimentally achieved better understanding among participants. This can be explained by the fact that a hybrid approach in written-medical-educational texts could represent a fusion between both: the terminology of medical literature and the context expressed in mother tongue. On a larger scale this approach, in our opinion, could harmonize both aforementioned aspects (interprofessional and patient-doctor communications).

As a matter of debate, an Indian-questionnaire-based study of 150 students and 25 teachers displayed that the majority of the participants considered English as a medium of instruction is not a problem [3]. In contrast, a previous study that included 114 Scandinavian family physicians, showing that those who read a review article in their mother tongue had the best retained medical information over those who read articles in other languages [22], this could be discussed in light of English vocabulary use in India in general education and communication.

In matter of preference, our results are in line with a previous study conducted in three universities in Sudan, revealing that most of the students and their instructors supported Arabic as a medium of instruction in medical colleges [32], and with another study conducted in a number of Saudi universities and showed that 23 (85%) science instructors who participated preferred to teach science subjects in Arabic [29]. For Saudi’s decision makers, English was the preferred as a language for teaching medicine but there was overall support for a future curriculum to be taught in Arabic once obstacles (such as translation costs) are overcome [9].

Albeit, in a previous Japanese report concerns were raised that Japanese medical students who are studying medicine in English for Specific Purpose could potentially confused medicalized English (colloquial English expressions, originally non-medical) with professional English medical terminology to describe patient’s conditions [33], a matter that can be resolved by the hybrid approach.

However, an Egyptian one-university study had shown that 31.5% of students were opposed to the idea of mother tongue curricula, and about 44% of them mentioned that learning in a foreign language posed a problem in understanding the scientific information, while nearly half of students translated most of the words in medical books to their mother language to facilitate studying [5]. The results of the aforementioned study may be discussed in light of the implicit all-or-none strategy proposed in their questionnaire. In comparison to this study; the evaluation in our study was focused solely on the written texts as a part of the educational process instead of doing an overall evaluation of the educational methods.

As aforementioned, the sample size was calculated on the basis of the last published data released by Syrian Ministry of Higher Education at the time of study design, the additional number (254) of Arabic-speaking participants from different Arabic and foreign universities represents an added value, in our opinion, as it allowed us to enlarge our sample size and evaluate potential differences in the preferable and most comprehensive written linguistic method between students from Syrian and non-Syrian Universities. However, Future studies with a larger number of participants from other countries might provide further comparison and explanation for these differences.

Conducting the survey online granted the advantage of reaching a non-conventionally-reachable cohort within a reasonable time and low expenses. These advantages of online surveys were discussed in previous studies [34–36], and of special interest regarding the difficulties and current circumstances of Syrian students. Furthermore, no funds or other resources were needed for the production and postage of a hardcopy survey, guaranteeing the independent and unbiased nature of this study. As a matter of discussion, the economic burden that lays on educational institutions to change some of their means of education and to adapt the system to a new linguistic approach is an important point [5, 6, 9], but seems potentially similar to the discussion of the implementation of mother language literacy programs, which was argued to be more cost-effective [37].

Finally, the doubt that a partially Arabic-based education may limit graduates’ ability to complete their studies internationally [5, 6, 9, 29] can be addressed as answered in previous studies conducted in the USA, showing that the performance of Syrian doctors in the ECFMG (Educational Commission for Foreign Medical Graduates) was equivalent to that of their peers, while the second showed that Damascus University was ranked the 7th highest among international universities that graduated the largest numbers of USA foreign licensed practitioners in 2016 [38, 39].

As shown before, better scientific understanding, in addition to enriching the students’ English knowledge could be achieved by bilingual medical education either by means of a bilingual medical course (BMC) [40, 41], selected medical activities [30, 42], or a hybrid system [9]. Our study managed to evaluate the written approach only. Thus, further studies are needed to evaluate other means of bilingual medical education.

It is of high importance that this study results should be investigated on other languages speaking populations in order to evaluate bilingual approach in educational written medical texts in different circumstances.

## Conclusion

To the best of our knowledge, this is the largest experimental study to assess a hybrid lingual-written approach in medical sciences. This aspect, along with the statistically significant results suggests that written medical texts with simplified mother tongue and English terminology may represent a viable method for medical education in non-English speaking populations. Further experimental-test-based studies from other universities in other countries to verify the applicability of these results and to test other educational methods in different non-English speaking populations are strongly recommended.

## Supplementary information

**Additional file 1: Appendix 1.** Questionnaire Structure. **Figure S1.** Study flow chart. **Table S1.** Test results of 1504 responders from Arabic language population (medical sciences students). **Table S2.** Right answers distribution; A. among 737 responders from Syrian Universities’ students who preferred Arabic language. And B. among 456 responders from Syrian Universities’ students who didn’t prefer Arabic language as a medium of medical sciences instruction. **Table S3.** Paragraph preference distribution; A. among 737 responders from Syrian Universities’ students who preferred Arabic language. And B. among 456 responders from Syrian Universities’ students who didn’t prefer Arabic language as a medium of medical sciences instruction. **Table S4.** Male/Female distribution; A. among 737 responders from Syrian Universities’ students who preferred Arabic language. And B. among 456 responders from Syrian Universities’ students who didn’t prefer Arabic language as a medium of medical sciences instruction. **Table S5.** Estimated Answering Time Distribution, and Distribution of Right Answers for the Paragraphs’ Questions in Less Than 1.5 Minute; A. among 737 responders from Syrian Universities’ students who preferred Arabic language. And B. among 456 responders from Syrian Universities’ students who didn’t prefer Arabic language as a medium of medical sciences instruction. (PDF 820 kb)

## Data Availability

The datasets used and/or analysed during the current study are available from the corresponding author on reasonable request.

## Abbreviation

ANOVA: Analysis of Variance
BMC: Bilingual Medical Course
CME: Continuing Medical Education
EBHC: Evidence-Based Health Care
ECFMG: Educational Commission for Foreign Medical Graduates
RCT’s: Randomized Control Trials
USA: United States of America
