# Supplementary material for: Language of written medical educational materials for non-English speaking populations: an evaluation of a simplified bi-lingual approach
Source: BMC Med Educ. 2019 Nov 11;19:418. doi: 10.1186/s12909-019-1846-x (PMC6849289; doi:10.1186/s12909-019-1846-x)
Supplement: Supplementary file 1 — Additional file 1: Appendix 1. Questionnaire Structure. Figure S1. Study flow chart. Table S1. Test results of 1504 responders from Arabic language population (medical sciences students). Table S2. Right answers distribution; A. among 737 responders from Syrian Universities’ students who preferred Arabic language. And B. among 456 responders from Syrian Universities’ students who didn’t prefer Arabic language as a medium of medical sciences instruction. Table S3. Paragraph preference distribution; A. among 737 responders from Syrian Universities’ students who preferred Arabic language. And B. among 456 responders from Syrian Universities’ students who didn’t prefer Arabic language as a medium of medical sciences instruction. Table S4. Male/Female distribution; A. among 737 responders from Syrian Universities’ students who preferred Arabic language. And B. among 456 responders from Syrian Universities’ students who didn’t prefer Arabic language as a medium of medical sciences instruction. Table S5. Estimated Answering Time Distribution, and Distribution of Right Answers for the Paragraphs’ Questions in Less Than 1.5 Minute; A. among 737 responders from Syrian Universities’ students who preferred Arabic language. And B. among 456 responders from Syrian Universities’ students who didn’t prefer Arabic language as a medium of medical sciences instruction. (PDF 820 kb) [file 12909_2019_1846_MOESM1_ESM.pdf]

Supplemental Digital Content

Appendix 1. Questionnaire Structure.
Figure 1. Study flow chart.
Table 1. Test results of 1504 responders from Arabic language population (medical sciences students).
Table 2. Right answers distribution; *A.* among 737 responders from Syrian Universities' students who preferred Arabic language. And *B.* among 456 responders from Syrian Universities' students who didn't prefer Arabic language as a medium of medical sciences instruction.
Table 3. Paragraph preference distribution; *A.* among 737 responders from Syrian Universities' students who preferred Arabic language. And *B.* among 456 responders from Syrian Universities' students who didn't prefer Arabic language as a medium of medical sciences instruction.
Table 4. Male/Female distribution; *A.* among 737 responders from Syrian Universities' students who preferred Arabic language. And *B.* among 456 responders from Syrian Universities' students who didn't prefer Arabic language as a medium of medical sciences instruction.
Table 5. Estimated Answering Time Distribution, and Distribution of Right Answers for the Paragraphs' Questions in Less Than 1.5 Minute; *A.* among 737 responders from Syrian Universities' students who preferred Arabic language. And *B.* among 456 responders from Syrian Universities' students who didn't prefer Arabic language as a medium of medical sciences instruction.

Appendix 1. Questionnaire Structure:

Page 1:
An informed consent was obtained from each participant in this page. A single response for each participant was achieved by adding an Email address section as a required field to proceed with the survey.

Page 2:
General information about the age, gender, current residence, college, university, educational qualification, and university grade point average were collected in this page.

Page3:
A Unified Medical Dictionary Arabic based scientific paragraph was presented in this page. The paragraph addressed the issue of obesity and its health complications. The final Arabic paragraph consisted of 110 words and it has been subjected to scientific auditing by three auditors.

Page 5:
The second paragraph that was included in this page was a combined English and simplified Arabic based paragraph without adherence to the Unified Medical Dictionary and it addressed the cardiovascular complications of type 2 diabetes mellitus. Essential English medical terms were paired with the Arabic medical terminologies within the text. The final paragraph consisted of 190 words and it has been subjected to scientific auditing by three auditors.

Page 6:
The English based paragraph was included in this page and it addressed Asthma. It consisted of 120 words. No modifications on the original text were performed.

The length and difficulty of the three provided texts were tested by 5 different individuals who possessed different scientific degrees, and later were adjusted in order to achieve a common average time span needed to read and comprehend the texts.

Page 4, 6, 8:
Each page contained three multiple-choice questions. The first question was written in Arabic, while the second one was written in English. These two questions assessed the respondents' understanding of the given paragraph. For each correct answer, a point was added to the final score (equal to 6 points in total) and the final score for each participant was calculated automatically and presented at the end of the survey.
The third question requested an estimation for the time needed to answer the previous two questions, as notified in the questionnaire instructions. They were required to select one of these three-time ranges: <1.5 minutes, 1.5-2.5 minutes and >2.5 minutes.

Page 9:
This page included two questions:
The first question: Do you prefer the first, second, or third paragraph's approach in teaching medical sciences? (First/second/third) were the answer options.
The second question: Do you support teaching medical sciences in the Arabic language? (Yes/no) were the answer options.

Page 10:
This was the last page of the questionnaire. It offered a direct link for respondents to view their final scores.

In order to complete the survey, respondents needed access to Google Chrome®, Mozilla Firefox®, Safari® or Internet Explorer®. We tested our questionnaire on several versions of web browsers running on different personal computers, tablets, and smartphones with various software systems, as Windows®, ios®, Android® and MacOS® systems.

Figure 1. Study Flow Chart

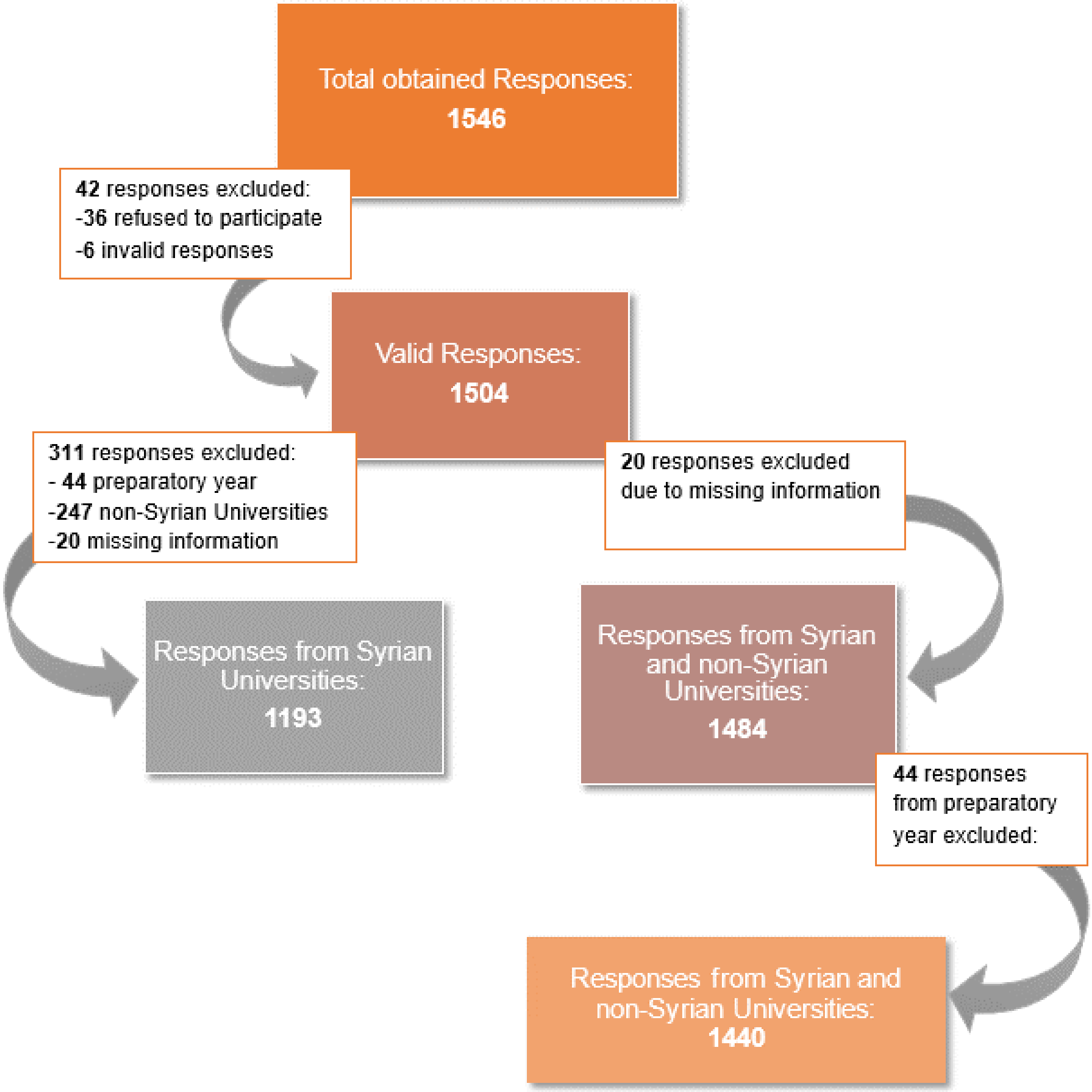

Table 1. Test results of 1504 responders from Arabic language population (medical sciences students).

	Question 1 (Arabic) Number (%) of Answers		Question 2 (English) Number (%) of Answers		Both Questions 1+2 Number (%) of Answers	
	Right Answers	Wrong Answers	Right Answers	Wrong Answers	Right Answers	Wrong Answers
**Paragraph 1 (Arabic)**	372 (24.7)	1132 (75.3)	607 (40.36)	897 (59.64)	165 (11)	1339 (89)
**Paragraph 2 (Hybrid)**	1274 (85)	230 (15)	1343 (89.3)	161 (10.7)	1173 (78)	331 (22)
**Paragraph 3 (English)**	411 (27.3)	1093 (72.7)	1260 (84)	244 (16)	346 (23)	1158 (77)

Table 2. Right answers distribution; *A.* among 737 responders from Syrian Universities' students who preferred Arabic language. And *B.* among 456 responders from Syrian Universities' students who didn't prefer Arabic language as a medium of medical sciences instruction.

Right Answers	*A.* Number(%) Of Right Answers Among Responders Who Preferred Arabic Language	*B.* Number(%) Of Right Answers Among Responders Who Didn't Prefer Arabic Language
**Arabic Questions**		
Right answers for all three Arabic questions	45 (6.1058)	26 (5.7017)
Right answer for the first Arabic question	174 (23.6092)	92 (20.1754)
Right answer for the second Arabic question	629 (85.3459)	387 (84.8684)
Right answer for the third Arabic question	203 (27.5441)	141 (30.921)
**English Questions**		
Right answers for all three English questions	217 (29.4436)	195 (42.7631)
Right answer for the first English question	269 (36.4993)	217 (47.5877)
Right answer for the second English question	651 (88.3310)	417 (91.4473)
Right answer for the third English question	587 (79.6472)	416 (91.228)
**All Questions**		
Right answers for all six questions	18 (2.4423)	11 (2.4122)
Right answers for the first paragraph's questions	73 (9.905)	40 (8.7719)
Right answers for the second paragraph's questions	576 (78.1546)	359 (78.728)
Right answers for the third paragraph's questions	161 (21.8453)	129 (28.2894)

Table 3. Paragraph preference distribution; *A.* among 737 responders from Syrian Universities' students who preferred Arabic language. And *B.* among 456 responders from Syrian Universities' students who didn't prefer Arabic language as a medium of medical sciences instruction.

Paragraph Preference	*A.* Number(%) Among Responders Who Preferred Arabic Language	*B.* Number(%) Among Responders Who Didn't Prefer Arabic Language
No clear preference	47 (6.3772)	21 (4.6053)
The first paragraph	117 (15.8752)	15 (3.2895)
The second paragraph	493 (66.8928)	128 (28.0701)
The third paragraph	80 (10.8548)	292 (64.0351)

Table 4. Male/Female distribution; *A.* among 737 responders from Syrian Universities' students who preferred Arabic language. And *B.* among 456 responders from Syrian Universities' students who didn't prefer Arabic language as a medium of medical sciences instruction.

**Male/Female**	***A.* Number(%) Among Responders Who Preferred Arabic Language**	***B.* Number(%) Among Responders Who Didn't Prefer Arabic Language**
Male	382 (51.8318)	228 (50)
Female	355 (48.1682)	228 (50)

Table 5. Estimated Answering Time Distribution, and Distribution of Right Answers for the Paragraphs' Questions in Less Than 1.5 Minute; *A.* among 737 responders from Syrian Universities' students who preferred Arabic language. And *B.* among 456 responders from Syrian Universities' students who didn't prefer Arabic language as a medium of medical sciences instruction.

**Answering time**	***A.* Number(%) Among Responders Who Preferred Arabic Language**	***B.* Number(%) Among Responders Who Didn't Prefer Arabic Language**
**Answering time for the first paragraph**		
X < 1.5 minute	592 (80.3257)	377 (82.6754)
1.5 < X < 2.5 minute	127 (17.320)	68 (14.9123)
2.5 < X minute	18 (2.4423)	11 (2.4123)
**Answering time for the second paragraph**		
X < 1.5 minute	607 (82.3609)	386 (84.6491)
1.5 < X < 2.5 minute	117 (15.8752)	63 (13.8158)
2.5 < X minute	13 (1.7639)	7 (1.5351)
**Answering time for the third paragraph**		
X < 1.5 min	515 (69.8779)	347 (76.0965)
1.5 < X < 2.5 min	170 (23.0665)	87 (19.0789)
2.5 < X	52 (7.0556)	22 (4.8246)
**Answering right in less than 1.5 minute**	***A.* Number(%) Among Responders Who Preferred Arabic Language**	***B.* Number(%) Among Responders Who Didn't Prefer Arabic Language**
Right answers for the first paragraph in < 1.5 minute	58 (7.8697)	33 (7.2368)
Right answers for the second paragraph in < 1.5 minute	487 (66.0787)	322 (70.614)
Right answers for the third paragraph in < 1.5 minute	116 (15.7395)	101 (22.1491)
Right answers for the three paragraphs in < 1.5 minute	11 (1.492)	5 (1.0965)
